# Epidural nalbuphine versus dexmedetomidine as adjuvants to bupivacaine in lower limb orthopedic surgeries for postoperative analgesia: a randomized controlled trial

**DOI:** 10.1186/s12871-023-02348-x

**Published:** 2023-12-06

**Authors:** Manal S. E. Farmawy, Sherif M. S. Mowafy, Rehab A. Wahdan

**Affiliations:** Department of Anesthesia, Intensive Care, and Pain Management, Faculty of Medicine, Zagazig University, Zagazig, Egypt

**Keywords:** Nalbuphine, Dexmedetomidine, Combined spinal-epidural anesthesia, Lower limb orthopedic surgeries, Lower limb surgeries, Postoperative analgesia

## Abstract

**Background:**

Administration of adjuvant drugs epidurally in combination with local anesthetics offers new dimensions in the management of postoperative pain. This study aimed to compare the addition of either nalbuphine or dexmedetomidine to epidural bupivacaine for postoperative analgesia in lower limb orthopedic surgeries under combined spinal-epidural anesthesia.

**Methods:**

This prospective randomized double-blind study included 69 patients scheduled for lower limb orthopedic surgeries. Anesthesia was started with 15 mg hyperbaric bupivacaine 0.5% intrathecally, and then an epidural bolus dose of 12 ml (10 ml 0.25% bupivacaine with 2 ml normal saline in group C, 2 ml (10 mg) nalbuphine in group N or dexmedetomidine 2 ml (100 µg) in group D was administered when sensory regression to T10. Postoperatively, when visual analogue scale (VAS) was ≥ 3, an epidural top-up dose of 8 ml (6 ml 0.25% bupivacaine plus 2 ml normal saline in group C, 2 ml (2 mg) nalbuphine in group N or 20 µg dexmedetomidine (2 ml) in group D was given. The primary outcome was to evaluate the duration of postoperative analgesia and secondary outcomes were any side effects and patient satisfaction.

**Results:**

The onset of epidural analgesia was 17.83 ± 2.53 versus 13.39 ± 1.27 versus 12.17 ± 1.27 min in groups C, N and D, respectively (*p* value < 0.001). The mean duration of analgesia was 241.3 ± 14.24 versus 318.38 ± 22.54 versus 365.87 ± 18.01 min in groups C, N and D, respectively (*p* value < 0.001). The mean sedation score was less in group C than group N and D (*P* < 0.001). The patient satisfaction score showed the lowest degree of satisfaction in group C (*p* value < 0.001). Top-up doses consumed and total analgesic requirements were lower in groups N and D than in group C. There was a statistically significant difference between the studied groups regarding VAS over time (*p* value < 0.001), intraoperative bradycardia (*p* value 0.029), and shivering (*p* value 0.029).

**Conclusion:**

The addition of either nalbuphine or dexmedetomidine to epidural bupivacaine was effective for postoperative analgesia in terms of onset, duration, and patient satisfaction with the superiority of dexmedetomidine over nalbuphine.

**Trial registration:**

Approval from the research ethics committee of the Faculty of Medicine, Zagazig University was obtained with the reference number (ZU-IRB#:7045-15-8-2021) and it was registered under clinicaltrials.gov (NCT05041270) on registration date 13/09/2021.

## Background

Surgical patients require effective intraoperative anesthesia and postoperative analgesia. Both spinal and epidural neuraxial blocks are widely used. Spinal anesthesia with a small dose of local anesthetic agent gives immediate and effective sensory and motor block, but its major side effects are hypotension and difficulty in controlling the level of the block [1]. Postoperative analgesia can be achieved by epidural anesthesia. Therefore, combined spinal epidural block (CSE) provides intense sensory and motor block with a long duration of analgesia extending to the postoperative period [2]. Neuraxial block provides analgesic effects by inhibiting nociceptive transmission from peripheral to central neuronal system, but this is limited by the short half-life of local anesthetics. Bupivacaine is an amide local anesthetic widely used for central and peripheral nerve block, and despite the relatively long duration of action, it is still insufficient for postoperative analgesia [3]. Adjuvant drugs are added to local anesthetics to prolong their duration and decrease their dose and side effects [4]. Nalbuphine, a derivative of 14-hydroxy morphine, is an analgesic with mixed kappa agonist and µ antagonist properties. Its potency is equal to that of morphine but exhibits a ceiling effect on respiratory depression [5]. Dexmedetomidine (DEX) is an imidazole compound highly selective α-2 adrenergic agonist with an affinity 8 times more specific than clonidine. It has sedative, sympatholytic, and analgesic effects that blunt cardiovascular responses both intraoperatively and postoperatively [6]. Dexmedetomidine causes manageable hypotension and bradycardia, but the advantage of this drug is the lack of opioid-related adverse effects [7].

Hence, we hypothesized that the addition of either nalbuphine or DEX to epidural bupivacaine could be effective in prolonging the duration of postoperative analgesia for patients undergoing lower limb orthopedic surgeries.

This study aimed to compare the addition of either nalbuphine or dexmedetomidine to epidural bupivacaine for the achievement of adequate postoperative analgesia in patients undergoing lower limb surgeries under combined spinal-epidural anesthesia.

## Methods

### Study design and population

This prospective randomized controlled clinical study was carried out on American Society of Anesthesiologists (ASA) physical status I and II (ASA I and II) patients scheduled for lower limb orthopedic surgeries of both sexes aged 21 – 60 years old at Zagazig University Hospitals during the period from October 1, 2021 to April 30, 2022 after approval from the institutional review board (Research ethical committee of Faculty of Medicine, Zagazig University) with reference number (ZU-IRB#7045-15-8-2021) and obtaining written informed consent from all patients before their enrollment. This study was registered under clinicaltrials.gov (NCT05041270) on registration date 13/09/2021.

Patients with known allergies to any of the study drugs or suffering from severe chronic diseases (cardiac, renal, hepatic, or neurological), presence of contraindications to neuraxial block, drug addiction or patient refusal were excluded from the study. If the spinal sensory block level did not reach T10 the patient was excluded from this study.

Preoperative history taking, clinical examination, routine investigations, and baseline measurements of patients (heart rate, mean arterial blood pressure (MAP), oxygen saturation) were recorded. Patients were instructed on how to express their pain, which was assessed by visual analog scale (VAS). The patient put a mark on a horizontal line at which 0 reads “no pain at all” at one end, and 10 means “worst imaginable pain” at the other end [8].

An intravenous line was secured, and patients were preloaded with (10 ml/kg) ringer lactate solution over 15–20 min. Patients sat on the operative table with their back curved and flexed forward. After cleaning and draping the back with a sterile sheet, the intervertebral space (L2-L3) was identified, and skin wheal was raised using a 26-gauge needle with 2% lidocaine. Tuohy needle number 18 was introduced and advanced slowly until the epidural space was identified by loss of resistance to air technique, with the bevel in the cephalic direction, an epidural catheter was inserted 5 cm into the epidural space and secured. Three milliliters of lidocaine (2%) with adrenaline (1/200,000) were injected through the epidural catheter as test dose. Then, L3-L4 intervertebral space was identified, and a 25-gauge spinal needle was introduced and 15 mg of 0.5% heavy bupivacaine was injected intrathecally. Surgery started under spinal anesthesia.

Patients were allocated randomly into three groups using computer generated randomization tables. A trained nurse who was blinded to the patient allocation and the study purpose prepared the study drugs.

#### Control group (Group C)

Epidural bolus dose of 12 ml (10 ml 0.25% bupivacaine plus 2 ml normal saline), followed by a top-up dose of 8 ml (6 ml 0.25% bupivacaine plus 2 ml normal saline).

#### Nalbuphine group (Group N)

Epidural bolus dose of 12 ml (10 ml 0.25% bupivacaine plus 10 mg nalbuphine (Nalbuphine-Sunny Pharmaceutical, Nalbuphine HCL 20 mg/ml) in 2 ml volume, top-up dose of 8 ml (6 ml 0.25% bupivacaine plus 2 mg nalbuphine in 2 ml volume) [2].

#### Dexmedetomidine group (Group D)

Epidural bolus dose of 12 ml (10 ml 0.25% bupivacaine plus 100 µg dexmedetomidine (Precedex™, Dexmedetomidine HCl 100 µg/mL, Pfizer Inc.) in 2 ml volume, top-up dose of 8 ml (6 ml 0.25% bupivacaine plus 20 µg dexmedetomidine in 2 ml volume) [9].

The level of sensory block was checked (for spinal then for epidural) by pinpricking with a 24G hypodermic needle at T10 dermatome midclavicular line using a 3-point scale: 0 = normal sensation, 1 = loss of sensation of pin prick (analgesia), 2 = loss of sensation of touch (anesthesia) [9].

The onset of sensory blockade injected intrathecally with maximal cephalic spread was assessed every 5 min for 30 min and then every 30 min. When sensory block regressed to T10 dermatome, epidural bolus dose was given according to each group. The onset, maximum level of sensory blockade and duration of epidural analgesia were recorded. The duration of analgesia from the time of epidural injection until VAS score ≥ 3 was recorded.

Monitoring of the patient’s vital signs (HR, MAP, and peripheral oxygen saturation) was recorded every 5 min for the first 30 min, after epidural bolus injection, and then every 30 min until the end of the operation. Hypotension (fall in mean blood pressure > 20% of baseline) treated by volume expansion or by incremental doses of IV ephedrine 3–6 mg. Bradycardia (heart rate < 60/min) was treated by 0.6 mg IV atropine.

Ramsey sedation score was used to assess the patient’s level of sedation (0–6) [10].

- 1 = Patient is anxious and agitated or restless, or both.
- 2 = Patient is cooperative, oriented, and tranquil.
- 3 = Patient responds to commands only.
- 4 = Patient exhibits brisk response to light glabellar tap or loud auditory stimulus.
- 5 = Patient exhibits a sluggish response to light glabellar tap or loud auditory stimulus.
- 6 = Patient exhibits no response.

The sedation score was recorded just before the initiation of epidural injection and 2 h postoperatively.

Postoperatively, patients were evaluated for pain every 2 h until 8 h and then every 4 h until 24 h. If VAS ≥ 3, injection of the top-up dose in the epidural catheter was performed according to each group, number and total doses were recorded. If pain persisted after 3 top-up doses with time interval of 30 min, ketorolac 30 mg IM was given, the timing and total dose required in the first 24 h were recorded. Patients completed 120 min after bolus injection in post anesthesia care unit (PACU) for monitoring and recording of any side effects postoperatively. 5-points Likert scale was used to evaluate patient satisfaction 24 h postoperatively, where 1 = very non satisfied, 2 = dissatisfied, 3 = neutral, 4 = satisfied, and 5 = highly satisfied.

The primary outcome of our study was the duration of postoperative analgesia defined by the time from epidural bolus injection till VAS ≥ 3 and secondary outcomes included the occurrence of any side effects related to the study drugs and patient satisfaction score.

### Sample size calculation

The mean duration of analgesia among patients receiving epidural dexmedetomidine was (305.2 ± 101.2) min [7] and among those receiving epidural nalbuphine is (380.3 ± 110.4) min [2] as an adjuvant to bupivacaine. Sample size was calculated by open Epi program to be 69 cases (23 cases in each group) with confidence level of 95% and power of test 80%.

### Statistical analysis

Data analysis was performed using the software SPSS (Statistical Package for the Social Sciences) version 26. Categorical variables were compared using the chi-square test, Fisher’s exact test and Monte Carlo test when appropriate. To compare ordinal data between groups, the Chi-square test for trend was used, and Kolmogorov–Smirnov (distribution-type) and Levene (homogeneity of variances) tests were used to verify assumptions for use in parametric tests. To compare quantitative data between more than two groups, one-way ANOVA (for normally distributed data) and the Kruskal–Wallis test (for nonnormally distributed data) were used. When the difference was significant, pairwise comparisons and Tukey’s HSD comparisons were used to detect differences between two individual groups. The level of statistical significance was set at *P* < 0.05. A highly significant difference was present if *p* ≤ 0.001.

## Results

**Fig. 1:**
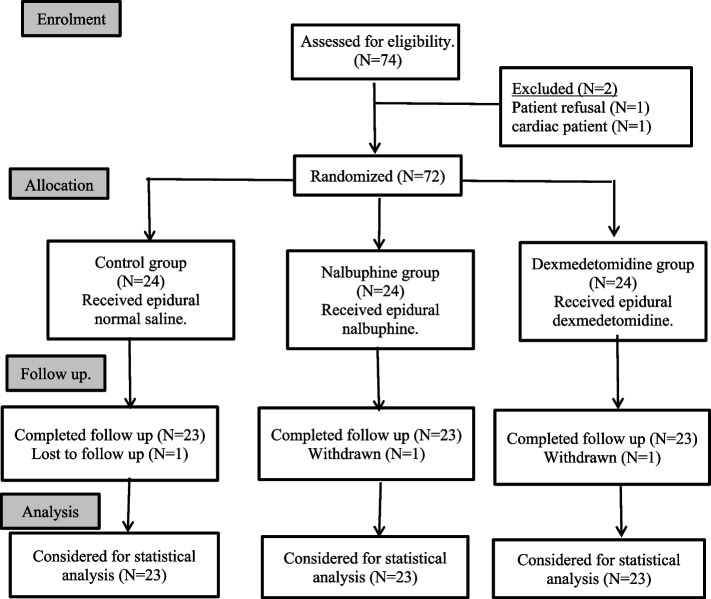
Flow chart of patients in the study groups

Seventy-four patients scheduled for lower limb orthopedic surgeries under CSE block were aligned in this study. From them 2 patients were excluded (1 patient refused to participate and the other was a cardiac patient), and 3 patients were withdrawn later (1 patient declined to complete the study follow-up, and 2 patients were complicated by post-dural puncture headache during the postoperative period); The net result 69 patients were enrolled and randomized as shown in flow chart (Fig. 1).

There was no statistically significant difference between the studied groups regarding age, sex, weight, height, BMI, ASA status, medical comorbidity as well as the type of surgical procedure (P > 0.05) (Table 1).

**Table 1:** Patients and clinical characteristics between the studied groups

Characteristics	Group C (*n* = 23)	Group N (*n* = 23)	Group D (*n* = 23)	*P* value
Age (years)	40.61 ± 10.97	39.91 ± 13.44	43.35 ± 11.15	0.588
Weight (kg)	76.52 ± 8.32	76.3 ± 8.42	76.09 ± 8.25	0.984
Height (cm)	170.43 ± 3.96	169.13 ± 3.89	168.7 ± 4.32	0.235
BMI (kg/m^2^)	26.49 ± 3.28	26.6 ± 3.01	26.67 ± 3.0	0.979
Sex Number (%)				
Male	19 (82.6%)	19 (82.6%)	16 (69.6%)	0.465
Female	4 (17.4%)	4 (17.4%)	7 (30.4%)	
ASA Number (%)				
ASA I	18 (78.3%)	15 (65.2%)	15 (65.2%)	0.54
ASA II	5 (21.7%)	8 (34.8%)	8 (34.8%)	
Medical Comorbidities Number (%)				
No	18 (78.3%)	15 (65.2%)	15 (65.2%)	0.971
DM	2 (8.7%)	2 (8.7%)	3 (13%)	
HTN	2 (8.7%)	5 (21.7%)	4 (17.4%)	
DM & HTN	1 (4.3%)	1 (4.3%)	1 (4.3%)	
Surgical type				
Tibial fracture	10 (43.5%)	8 (34.8%)	8 (34.8%)	> 0.999
Femur fracture	5 (21.7%)	5 (21.7%)	6 (26.1%)	
DHS	5 (21.7%)	5 (21.7%)	5 (21.7%)	
Pott’s fracture	3 (13%)	5 (21.7%)	4 (17.4%)	

Data are expressed as the mean ± SD and number (precent)

One-way ANOVA test, chi-square test, Monte Carlo test

*Group C* Control group, *Group N* Nalbuphine group, *Group D* Dexmedetomidine group, *n* Total number of subjects in each group, *BMI* Body mass index, *DM* Diabetes mellitus, *HTN* Hypertension, *DHS* Dynamic hip screw

*P* < 0.05 was considered significant

Epidural onset, it was statistically highly significant longer in control group than in the two other groups. The epidural duration of analgesia was shorter in the control group than in groups N and D, and the longest duration was obtained in group D. There was statistically nonsignificant difference between the studied groups regarding the level of sensory block. The level of sedation was significantly higher in group D (Table 2).

**Table 2:** Comparison between the studied groups regarding epidural onset, duration, level of sedation and sensory block level

Variables	Group C (*n* = 23)	Group N (*n* = 23)	Group D (*n* = 23)	*P* value
Epidural onset (min)	17.83 ± 2.53^a^	13.39 ± 1.27	12.17 ± 1.27	< 0.001
Epidural duration (min)	241.3 ± 14.24	318.38 ± 22.54	365.87 ± 18.01^b^	< 0.001
Level of sedation Number (%)				
1	2 (8.7%)	2 (8.7%)	1 (4.3%)	< 0.001
2	21 (91.3%)	14 (60.9%)	9 (39.1%)	
3	0 (0%)	7 (30.4%)	9 (39.1%)	
5	0 (0%)	0 (0%)	4 (17.4%)^c^	
Sensory block Number (%)				
T6	10 (43.5%)	8 (34.8%)	12 (52.2%)	0.293
T8	13 (56.5%)	15 (65.2%)	11 (47.8%)	

One-way ANOVA test, chi-square test, Monte Carlo test

*Group C* control group, *Group N* nalbuphine group, *Group D* dexmedetomidine group, *n* total number of subjects in each group

*P* < 0.05 was considered statistically significant

*P* ≤ 0.001 is highly statistically significant

^a^Epidural onset was highly significantly longer in the control group

^b^Epidural analgesia duration was highly significant longer in group D

^c^Level of sedation was significantly higher in group D

**Fig. 2:**
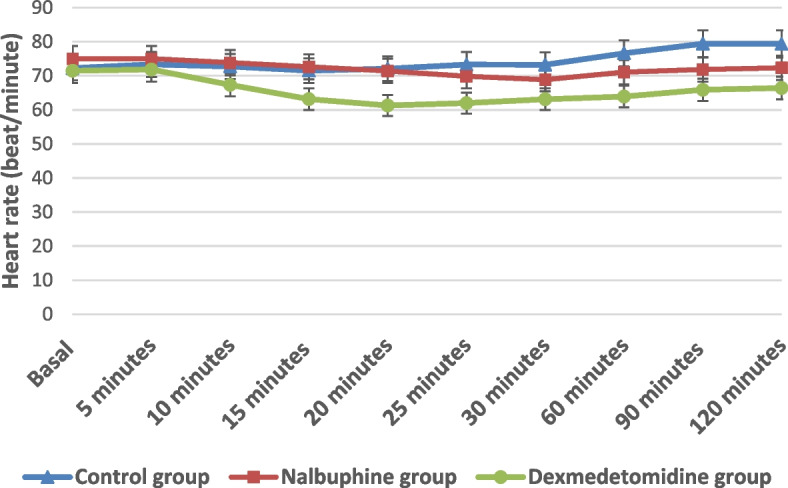
Comparison between the studied groups regarding heart rate (beat/min)

**Fig. 3:**
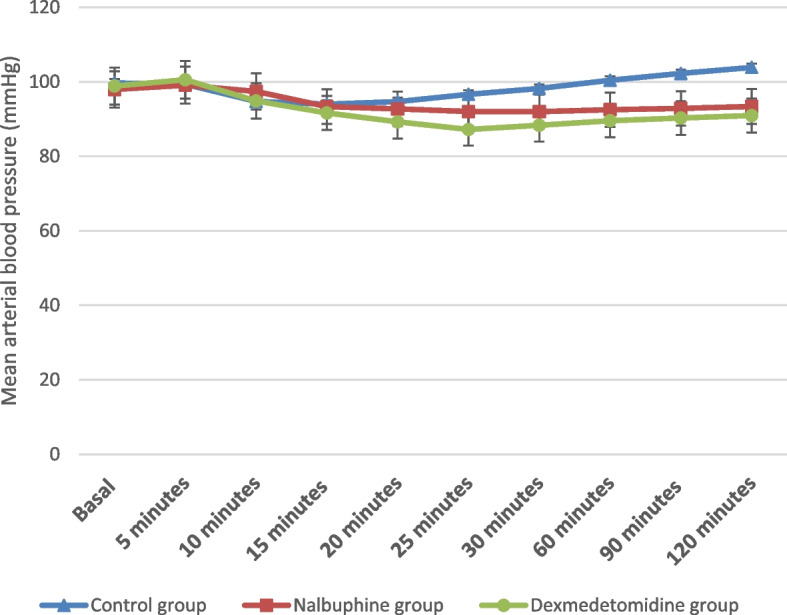
Comparison between the studied groups regarding mean arterial blood pressure (mmHg)

There was statistically significant difference between the studied groups regarding heart rate at 10, 15, 20, 25, 30, 60, 90 and 120 min. On doing Tukey’s HSD comparison for heart rate at 10, 15 and 20 min, the difference was significant between the dexmedetomidine group and each other group, and it was nonsignificant at baseline and 5 min (Fig. 2). There was a statistically nonsignificant difference between the studied groups regarding MAP at baseline, and at 5, 10, 15 and 20 min, but the difference was significant at 25, 30, 60, 90 and 120 min. By performing Tukey’s HSD comparison for MAP at 30, 60, 90 and 120 min, the difference was significant between the control group and each other group, while concerning MAP at 25 min, the difference was significant between the dexmedetomidine and control groups (Fig. 3).

**Fig. 4:**
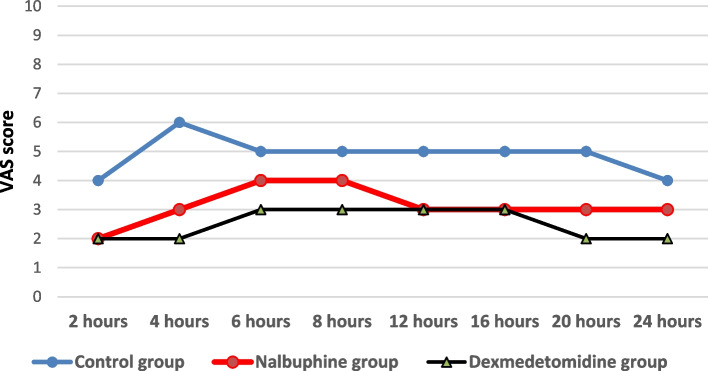
Comparison between the studied groups regarding VAS. VAS = Visual analog scale

When evaluating the VAS over time, there was a statistically significant difference between the studied groups. On performing pairwise comparisons, the difference was significant between the control group and each other group (Fig. 4).

Regarding patient satisfaction, the difference between the studied groups was statistically significant where high degrees of satisfaction were obtained in group D patients and the larger percentage of control group patients showed lowest degrees of satisfaction as well as the need for epidural top up doses showed statistically highly significant difference between the three studied groups and between each two-group with more doses were needed in group C (*p* < 0.001) and regarding persistence of pain after 3 top up doses with the need for ketorolac to treat this pain, it was statistically significant different between the three studied groups where it was highly needed with larger doses in control group when compared to nalbuphine and dexmedetomidine groups (Table 3).

**Table 3:** Comparison between the three studied groups regarding patient satisfaction score, epidural top-up doses needed, and the need for ketorolac

Variables	Group C (*n* = 23)	Group N (*n* = 23)	Group D (*n* = 23)	*P* value
Satisfaction Score Number (%)				
3	17 (73.9%)	2 (8.7%)	2 (8.7%)	< 0.001
4	6 (26.1%)	16 (69.5%)	11 (47.8%)	
5	0 (0%)	5 (21.7%)	10 (43.5%)^a^	
Top up doses needed. Number (%)				
3	0 (0%)	1 (4.3%)	9 (39.1%)	< 0.001
4	1 (4.3%)	11 (47.8%)	13 (56.5%)	
5	9 (39.1%)	11 (47.8%)	1 (4.3%)	
6	12 (52.2%)^b^	0 (0%)	0 (0%)	
7	1 (4.3%)^b^	0 (0%)	0 (0%)	
Ketorolac needed. Number (%)				
No	0 (0%)	11 (47.8%)	14 (60.9%)	< 0.001
30 mg	12 (52.2%)^c^	9 (39.1%)	9 (26.1%)	
60 mg	11 (47.8%)^c^	3 (13%)	3 (13%)	

Chi-square test

*Group C* control group, *Group N* nalbuphine group, *Group D* dexmedetomidine group, *n* total number of subjects in each group

*P* < 0.05 was considered statistically significant

*P* ≤ 0.001 is highly statistically significant

^a^High degrees of satisfaction were obtained among group D patients

^b^The need for epidural top-up doses was significantly higher among control group patients than among other groups

^c^Ketorolac was needed at larger doses in group c than in groups N and D

Regarding adverse effects, the incidence of bradycardia significantly differed between the nalbuphine and dexmedetomidine groups, and the shivering incidence significantly differed between the nalbuphine and control groups. Additionally, there was a statistically significant difference between the studied groups regarding hypotension and nausea/vomiting incidence. Moreover, the need for drugs to treat these adverse effects was significantly different between the studied groups, as the need for ephedrine was significantly higher in the control group than in the other two groups. Additionally, atropine needs were significantly different between group N and group D (1 patient (4.3%) versus 8 patients (34.8%) in groups N and D, respectively) (Table 4).

**Table 4:** Comparison between the three studied groups regarding adverse effects and medications given

Variables	Group C (*n* = 23)	Group N (*n* = 23)	Group D (*n* = 23)	*P* value
Hypotension Number (%)	6 (26.1%)	5 (21.7%)	4 (17.4%)	0.774
Bradycardia Number (%)	6 (26.1%)	1 (4.3%)	8 (34.8%)^a^	0.029
Shivering Number (%)	8 (34.8%)^b^	1 (4.3%)	2 (8.7%)	0.029
Nausea & vomiting Number (%)	6 (26.1%)	2 (8.7%)	2 (8.7%)	0.116
Medications given				
Ephedrine Number (%)				
No	11 (47.8%)	18 (78.3%)	19 (82.6%)	0.014
3 mg	6 (26.1%)^c^	3 (13%)	3 (13%)	
6 mg	6 (26.1%)^c^	2 (8.7%)	1 (4.3%)	
Atropine 0.6 mg Number (%)	2 (8.7%)	1 (4.3%)	8 (34.8%)^d^	0.029

Chi-square test

*Group C* control group, *Group N* nalbuphine group, *Group D* dexmedetomidine group, *n* total number of subjects in each group

*P* < 0.05 was considered statistically significant

*P* ≤ 0.001 is highly statistically significant

^a^Bradycardia was significantly common among group D patients

^b^Shivering was significantly common among group C patients

^c^The need for Ephedrine was significantly high in group C

^d^The need for atropine was significantly high in group D

## Discussion

Opioids and alpha 2 agonists as epidural adjuvants were found to produce potent analgesic effects. These adjuvants not only improve the onset and duration of the block, but also reduce the need for other enteral or parental analgesics and gain better patient satisfaction [11].

The current study investigated the addition of either nalbuphine or DEX to epidural bupivacaine on the duration of postoperative analgesia in patients scheduled for lower limb orthopedic surgeries. Our results proved that the addition of both nalbuphine and DEX faster the onset and prolonged the duration of analgesia than bupivacaine alone. The time required for the onset of sensory block was (12.17 ± 1.27, 13.39 ± 1.27, and 17.83 ± 2.53 min. in DEX, nalbuphine, and control groups respectively) and the duration of epidural analgesia was 365.87 ± 18.01 min in DEX group versus 318 > 38 ± 22.54 min in nalbuphine group and 241.3 ± 14.24 min in control group. Therefore, the fastest onset and longest duration were obtained in the DEX group.

Since, the discovery of opioid receptors in the brain and spinal cord, the postoperative analgesia field has changed with several opioids have been studied as adjuvants to local anesthetics in order to minimize side effects and prolong the duration of both intraoperative and postoperative analgesia. Nalbuphine is an opioid with agonistic action at kappa and antagonistic action at µ receptors that was found to provide adequate analgesia in visceral nociception and to improve postoperative analgesia [2, 12]. Catrath et al., conducted a comparative study of epidural bupivacaine with nalbuphine versus bupivacaine with tramadol for postoperative analgesia in lower limb orthopedic surgeries under CSE anesthesia. They found that the analgesia duration was prolonged and sedation score was higher in tramadol group than in nalbuphine group, but fewer side effects and higher patient satisfaction scores were observed in nalbuphine group. They concluded that both nalbuphine and tramadol were effective for postoperative analgesia when used epidurally. However, nalbuphine was better with fewer complications e.g., nausea, vomiting and sedation and better patient satisfaction [2].

DEX could be an opioid-sparing epidural adjuvant that produces its analgesic effect by hyperpolarization of post-synaptic dorsal horn neurons and inhibiting the release of C fibers transmission [13]. Eskandar and Ebeid investigated the effects of epidural DEX with low volume bupivacaine in patients undergoing elective total knee replacement and they recommended DEX as an ideal epidural adjuvant to bupivacaine for postoperative analgesia because it decreases both epidural local anesthetic volume and postoperative analgesic requirements with stable cardiorespiratory parameters [14]. In another study, Batham et al. compared the addition of fentanyl or dexmedetomidine to epidural bupivacaine for patients who underwent lower limb orthopedic surgeries, and they observed that DEX had a significantly early onset of sensory anesthesia, and prolonged postoperative analgesia with marked decrease in postoperative pain scores [15]. These conclusions agreed with those of Soliman et al. [16] and Paul et al. [9], who reported improved postoperative analgesia and decreased need for postoperative opioids with the addition of DEX. Additionally, Emam et al., concluded that DEX was preferable as an epidural adjuvant compared to fentanyl for postoperative analgesia after abdominal surgeries [17].

In the present study, the number of top-up doses and the VAS scores were also significantly lower in DEX group patients, and the persistence of pain after 3 top-up doses with the need for ketorolac was significantly different between the three groups where it was highly needed with larger doses in control group when compared to nalbuphine and DEX groups.

However, in this study, the level of sedation was higher in DEX group, which could be attributed to the highly selective α-2 adrenergic agonist action with sedative, sympatholytic and analgesic effects, while nalbuphine is a µ receptor antagonist that has a ceiling effect on sedation where additional sedation does not increase with dose increasing [18]. Consistent with our results, Salgado et al., found that patients in dexmedetomidine group were more sedated with lower bispectral values compared to the control group [19]. The sedative and analgesic effects of DEX have been proven in numerous previously published studies [14, 20–22].

Regarding adverse effects, the incidence of bradycardia in our study was significantly common in DEX group patients which is explained by the DEX central action decreasing the sympathetic outflow and norepinephrine release, as found in several previous studies [22–24].

Hypotension was observed more often in control group patients however it was not statistically significant between groups. This shows that the epidural administration of DEX and nalbuphine in their respective doses we opted to use in our study were safe in providing hemodynamically stable perioperative period. Additionally, the incidence of adverse effects and complications in our study were minimal and managed appropriately and because of fewer side-effects in DEX group, high degrees of satisfaction were obtained in this group.

Therefore, our findings support that the addition of either nalbuphine or dexmedetomidine to epidural bupivacaine was effective for postoperative analgesia and the earlier onset as well as the longest analgesic duration with fewer side effects and higher patient satisfaction scores were observed in DEX group. This is in line with the results of Khobragade et al., who compared the addition of DEX and nalbuphine to bupivacaine in epidural anesthesia for infraumbilical and lower limb surgeries and found that the onset of sensory block was significantly earlier, and the analgesia duration was significantly prolonged in DEX group versus nalbuphine group (10.06 ± 4.42 versus 13.88 ± 7.83 min., and 353.86 ± 51.36 versus 295.28 ± 65.95 min for the onset and duration of analgesia respectively) with stable hemodynamics and fewer side effects in DEX patients concluding that DEX is a better adjuvant than nalbuphine for epidural anesthesia [25]. Also, Lakshmi et al. in a randomized double-blind placebo-controlled trial compared the efficacy of epidural nalbuphine versus DEX on spinal anesthesia characteristics in patients who underwent lower limb orthopedic surgeries reporting that in terms of earlier onset and longer duration of both sensory and motor blocks, longer postoperative analgesia with useful intraoperative sedation DEX as an epidural adjuvant was found to be better than nalbuphine [26].

DEX and nalbuphine were also investigated as epidural adjuvants to 0.25% bupivacaine for labor analgesia by El Fawal and his colleagues concluding that both provided satisfactory labor analgesia without severe side effects and that DEX has a faster onset than nalbuphine [27].

Despite the aforementioned studies supporting the epidural use of either DEX or nalbuphine with no harm, up to the best of authors’ knowledge DEX and nalbuphine are currently not approved by FDA for epidural administration. Nalbuphine was approved by FDA for moderate to severe pain that requires an opioid agent when other alternative treatments have been inadequate in 1998 and DEX was initially approved in 1999 for short-term sedation (< 24 h) in ICUs and in 2022, FDA approved its sublingual formulation for acute treatment of agitation associated with schizophrenia or bipolar I or II disorder in adults. Although they are widely used in anesthesia as an adjuvant in neuraxial blocks as well as the outcome of most studies is encouraging, FDA did not approve their off-label use as an epidural adjuvant. Several nalbuphine non-FDA approved uses do exist, such as treatment of labor pain, opioid-induced respiratory depression, opioid-induced urinary retention, and pruritus linked to neuraxial opioid administration as well as it is increasingly used during neuraxial blocks due to its high safety. Also, epidural DEX was demonstrated in various studies to be well tolerated. Hence, FDA approval is required for the uncontroversial use of both DEX and nalbuphine in anesthesia practice.

The limitations of our study are the relatively small sample size of patients who were included, the subjectivity of VAS for pain assessment with a variable level of understanding between patients, and there are no available comparisons of equipotent dosing of epidural nalbuphine versus dexmedetomidine.

## Conclusion

In conclusion, the addition of either nalbuphine or dexmedetomidine to epidural bupivacaine was effective for postoperative analgesia. The fastest onset and longer analgesic duration with fewer side effects as well as higher patient satisfaction was observed with dexmedetomidine. Therefore, dexmedetomidine is a better epidural adjuvant than nalbuphine for postoperative analgesia in patients undergoing lower limb orthopedic surgeries.

## Data Availability

The data used and analyzed during our study are available from the corresponding author on reasonable request.

## Abbreviations

CSE: Combined Spinal-Epidural Block
ASA: American Society of Anesthesiology
DEX: Dexmedetomidine
MAP: Mean arterial pressure
VAS: Visual analog scale
PACU: Post anesthesia care unite
